# Omega-3 fatty acid improves the clinical outcome of hepatectomized patients with hepatitis B virus (HBV)-associated hepatocellular carcinoma

**DOI:** 10.7555/JBR.26.20120058

**Published:** 2012-10-23

**Authors:** Zhengshan Wu, Jianjie Qin, Liyong Pu

**Affiliations:** a Liver Transplantation Center, the First Affiliated Hospital, Nanjing Medical University, Nanjing, Jiangsu 210029, China;; b Key Laboratory of Living Donor Liver Transplantation, Ministry of Public Health, Nanjing, Jiangsu 210029, China.

**Keywords:** omega-3 fatty acids, hepatectomy, total parenteral nutrition, hepatocellular carcinoma

## Abstract

Omega-3 fatty acid supplemented total parenteral nutrition improves the clinical outcome of patients undergoing certain operations; however, its benefits for patients with hepatitis type B virus (HBV)-associated hepatocellular carcinoma (HCC) who have undergone hepatectomy are still not clear. The aim of this study was to evaluate the effect of omega-3 fatty acid supplemented total parenteral nutrition on the clinical outcome of patients with HBV-associated HCC who underwent hepatectomy at our institution. A total of 63 patients with HBV-associated HCC who underwent hepatectomy were included in this study. These patients were randomly assigned to receive standard total parenteral nutrition (the control group, *n* = 31) or omega-3 fatty acid supplemented total parenteral nutrition (the omega-3 fatty acid group, *n* = 32) for at least 5 d. The study endpoints were the occurrence of infection-related complications, recovery of liver function and length of hospital stay. The results showed that the omega-3 fatty acid group had a lower infection rate (omega-3 fatty acid, 19.4% vs control, 43.8%, *P* < 0.05), a better liver function after hepatectomy: alanine transaminase (omega-3 fatty acid, 48.23±18.48 U/L vs control, 73.34±40.60 U/L, *P* < 0.01), aspartate transaminase (omega-3 fatty acid, 35.77±14.56 U/L vs control, 50.53±24.62 U/L, *P* < 0.01), total bilirubin (omega-3 fatty acid, 24.29±7.40 mmol/L vs control, 28. 37±8.06 mmol/L, *P* < 0.05) and a shorter length of hospital stay (omega-3 fatty acid, 12.71±2.58 d vs control, 15.91±3.23 d, *P* < 0.01). The serum contents of IL-6 (omega-3 fatty acid, 23.98±5.63 pg/mL vs control, 35.55±7.5 pg/mL, *P* < 0.01) and TNF-α (omega-3 fatty acid, 4.43±1.22 pg/mL vs control, 5.96±1.58 pg/mL, *P* < 0.01) after hepatectomy were significantly lower in the omega-3 fatty acid group than those of the control group. In conclusion, administration of omega-3 fatty acid may reduce infection rate and improve liver function recovery in HBV-associated HCC patients after hepatectomy. This improvement is associated with suppressed production of proinflammatory cytokines in these patients.

## INTRODUCTION

Lipids are key energy sources in critically ill patients due to their high energy density, low osmolarity, and preferred utilization during the systemic inflammatory response. Benefits of omega-3 fatty acids have been observed in humans and in animal models of diabetes, obesity, cancer, and cardiovascular diseases[1]–[5]. Thus, it is important to promote the consumption of omega-3 fatty acids.

Recent studies have indicated that omega-3 fatty acids are safe for cancer patients and play a very important role for patients who need total parenteral nutrition (TPN)[6]–[10]. Using a high-dose short-term infusion of omega-3 fatty acid lipid emulsion may cause rapid immunologic changes, through which the effects on the endotoxin-induced stress response may be achieved. In this randomized controlled study, we evaluated the effect of omega-3 fatty acid-supplemented TPN on the clinical outcomes of patients with hepatitis B virus (HBV)-associated hepatocellular carcinoma (HCC) after hepatectomy.

## SUBJECTS AND METHODS

### Patients

In this prospective, randomized, controlled single center study, 69 consecutive patients with liver cancer undergoing hepatectomy were prospectively enrolled from January 2007 and December 2008. All the enrolled patients underwent hepatectomy due to HCC. The inclusion criteria were preoperative liver function of Child-Pugh A and serum HBV-DNA less than 1,000 copies/L. Eligible patients underwent hepatectomy were assigned to receive TPN for at least 5 d with or without omegat-3 fatty acid supplment. Six patients were excluded from the study because they received TPN for less than 5 d. The major exclusion criteria were severe hemorrhage, unstable diabetes mellitus, persistent hemodynamic failure (systolic blood pressure <90 mmHg), and allergic reactions to fish or egg proteins. There were no remarkable differences between the two groups in gender, ages, diagnosis, warm ischemia time, body mass index, liver function, blood loss and duration of surgery (***Table 1***).

The study protocol was approved by the local institutional review board at the authors' affiliated institutions and informed consent was obtained from each subject or his or her legal surrogates.

### Interventions

Patients were randomized by computer-derived block randomization. The pharmacist was the only person who was aware of the randomization list. Both the patients and the investigators were unaware of the infused drug. All the patients received TPN consisting of amino acids, glucose, a soybean oil emulsion or an omega-3 lipid emulsion supplement (Omegaven, Fresenius-Kabi; Omegaven is comprised 10% pure omega-3 fatty acids,) for longer than 5 d via a central venous catheter. The TPN emulsion was freshly prepared in the hospital pharmacy and labeled with patient and study numbers, the name of the investigator, and the date of the preparation. Omega-3 fatty acids were added to standard TPN. Both groups of patients received isonitrogenous (0.24 g/kg·d) and isocaloric (35 kcal/k·d) regimens supplied as constant intravenous infusion via central veins. The supply was administered for 5 to 7 d (the control group, 5.8±0.6 d; the omega-3 fatty acid group, 5.6±0.4 d) as clinically indicated, as follows: 1.5 g/kg·d of amino acids (Novamin), 3.5 g/kg·d of glucose, 1.5 g/kg·d of lipid emulsion (Lipovenos or Omegaven). Standard vitamins (Soluvit), electrolytes and trace elements (Addamel) were supplied according to the protocol of our hospital. Insulin therapy was started according to the level of blood glucose when indicated (glucose:insulin 4:1).

### Laboratory studies

The parameters alanine transaminase (ALT), aspartate transaminase (AST), total bilirubin (TBIL), and albumin (ALB) were quantified by the Department of Clinical Chemistry, the First Affiliated Hospital of Nanjing Medical University (Nanjing, China) according to standard procedures. Serum IL-6 and TNF-α concentrations were determined using the enzyme-linked immuno-sorbent assay (ELISA) kit supplied by Biosource International (CA, USA).

All patients were followed up for 6 months after the discharge from the hospital.

**Table 1:** Characteristics of patients in the study

Characteristics	Omega-3 fatty acid group (*n* = 31)	Control group (*n* = 32)	*P*
Gender (male/female)	25/6	25/7	0.805
Age (years)	52.00±7.90	52.16±8.31	0.939
Body mass index (BMI)	22.47±1.80	22.64±1.63	0.702
Duration of operation (h)	03.57±0.98	03.64±0.93	0.754
Warm ischemia time (min)	11.52±4.86	11.56±4.09	0.967
Blood loss (mL)	.0344±146.	.0341±137.	0.942
Volume of resection (mL)	.0179±106.	.0187±125.	0.786

**Table 2:** Clinical outcomes of the patients in the study

Variables	Omega-3 fatty acid group (*n* = 31)	Control group (*n* = 32)	*P*
Nosocomial infections	6	14	< 0.05
LOS (d)	12.71±2.58	15.91±3.23	< 0.01
Mortality (6 months)	0	0	> 0.05

LOS: the length of hospital stay.

### Statistical analysis

The calculation was based on the results of the previous clinical trial available at the time when the trial was designed. The calculated sample size reached the number of 63 evaluable patients. To take into account some dropouts, the trial was planned initially to include 70 patients. Patients with less than 5 d of TPN were not eligible for efficacy evaluation. Finally, 63 patients were included. Statistical analysis was performed by SPSS (Version 16.0, Chicago, IL.China). Quantitative data were presented as arithmetic mean±SD. Quantitative variance was analyzed by Student's *t*-test and qualitative variance was analyzed by chi-square test. *P* < 0.05 was considered statistically significant.

## RESULTS

### Patients receiving omega-3 fatty acid in TPN after hepatectomy had a lower nosocomial infection rate

Patients receiving omega-3 fatty acid had a significant lower nosocomial infection rate (omega-3 fatty acid, 6/31, 19.4% vs control, 14/32, 43.8%, *P* < 0.05) (***Table 2***). The decrease in nosocomial infections in patients receiving omega-3 fatty acid was mainly due to reduced incidence of pneumonia (omega-3 fatty acid, 4/32, 12.5% vs control, 11/31, 35.48%, respectively). The overall incidence of other infections (surgical wound, urinary or intravenous catheter infection) was similar in the omega-3 fatty acid and control group with an average of 2 and 3 episodes.

The six-month outcome was evaluated for all patients through reviewing hospital charts or contacting the practitioners. The length of hospital stay (omega-3 fatty acid, 12.71±2.58 d vs control, 15.91±3.23 d, *P* < 0.01) (***Table 2***) was shorter in the omega-3 fatty acid group than the control group. The six-month mortality was not different between the two groups (***Table 2***).

### Patients receiving omega-3 fatty acid in TPN after hepatectomy had a faster recovery of liver function

The liver function tests were significantly different between the two groups after hepatectomy on postoperative d 6: ALT (omega-3 fatty acid, 48.23±18.48 U/L vs control, 73.34±40.60 U/L, *P* < 0.01), AST (omega-3 fatty acid, 35.77±14.56 U/L vs control, 50.53±24.62 U/L, *P* < 0.01), TBIL (omega-3 fatty acid, 24.29±7.40 mmol/L vs control, 28.37±8.06 mmol/L, *P* < 0.05) (***Table 3***). But there was no difference between the two groups in plasma albumin concentrations (omega-3 fatty acid, 39.84±3.87 g/L vs 38.69±3.91 g/L, *P* = 0.245) (***Table 3***).

**Table 3:** Liver functions of patients in the study

Variables	Omega-3 fatty acid group (*n* = 31)	Control group (*n* = 32)	*P*
Pre-operation:			
ALT	028.39±14.84	029.22±17.22	< 0.838
AST	025.84±13.04	025.69±13.24	< 0.964
TBIL	17.58±4.96	16.81±4.45	< 0.520
ALB	40.48±4.25	40.44±3.78	< 0.964
POD 6:			
ALT	048.23±18.48	073.34±40.60	< 0.010
AST	035.77±14.56	050.53±24.62	< 0.010
TBIL	24.29±7.40	28.37±8.06	< 0.050
ALB	39.84±3.87	38.69±3.91	< 0.245

ALB: albumin; ALT: alanine transaminase; AST: aspartate transaminase; TBIL: total bilirubin.

**Table 4:** Serum IL-6 and TNF-α of the patients in the study

Variables	Omega-3 fatty acid group (*n* = 31)	Control group (*n* = 32)	*P*
Pre-operation:			
IL-6	13.45±3.21	013.04±3.83	< 0.647
TNF-α	03.69±1.41	003.72±1.37	< 0.935
POD 6:			
IL-6	23.98±5.63	35.55±7.5	< 0.010
TNF-α	04.43±1.22	005.96±1.58	< 0.010

POD: post operation day; IL-6: interleukin-6; TNF-α: tumor necrosis factor-α.

### Patients receiving omega-3 fatty acid in TPN after hepatectomy had lower serum levels of proinflammatory cytokines

Because IL-6 and TNF-α are associated with acute inflammation, we measured the serum contents of IL-6 and TNF-α in the two groups. Although there were no differences in the two groups before hepatectomy, serum IL-6 content (omega-3 fatty acid, 23.98±5.63 pg/mL vs 35.55±7.5 pg/mL, *P* < 0.01) and TNF-α content (omega-3 fatty acid, 4.43±1.22 pg/mL vs 5.96±1.58 pg/mL, *P* < 0.01) in the dmega-3 fatty acid group after hepatectomy on postoperative d 6 were significantly lower than the control group (***Table 4***).

## DISCUSSION

In addition to caloric support, lipid emulsions have the capacity to modify the immune response. Omega-3 fatty acids are recognized for their ability to modify leukocyte activity, alter lipid-mediator generation, and to modulate cytokine release[11]. A recent meta-analysis showed that use of Omega-3 fatty acid lipid emulsions which are rich in omega-3 fatty acids may reduce ventilation time, mortality, antibiotic demand, and length of hospital stay in surgical patients[12]. Omega-3 fatty acids contained in fish oil have beneficial properties of reduction of inflammatory reaction and immunological modulation[13].

In this single center, prospective, double-blind trial, omega-3 fatty acid -supplemented TPN improves the clinical outcome after hepatectomy. The two groups have similar demographic and baseline characteristics at inclusion. The complication rate in the control group is higher than that of the treatment group, which is consistent with previously published results[14],[15]. Omega-3 fatty acid-treated liver cancer patients clearly benefit from omega-3 fatty acid supplementation[16]. In the present study, omega-3 fatty acid-supplemented TPN was associated with a lower incidence of infectious events. The result is in accordance with previous study revealing the reduction of infectious events observed after administration of omega-3 fatty acid enteral nutrition to trauma patient[17]. This study suggests that omega-3 fatty acid-enriched nutrition reduces infectious complications in patients after hepatectomy.

The reduction of infectious complications is mainly due to a lower incidence of pneumonia as previously reported with the use of omega-3 fatty acid-enriched enteral nutrition[18]. Mechanisms accounting for these effects are reduced levels of PGE2 and inducible NO synthase as well as an increased lipid peroxidation. Furthermore, omega-3 fatty acid is capable of down-regulating the production of a number of mediators such as IL-1, IL-6, TNF-α and proteolysis-inducing factor[16],[19]. In our study, serum IL-6 and TNF-α levels in the omega-3 fatty acid group after hepatectomy on postoperative day 6 were significantly lower than the control group, revealing that omega-3 fatty acids may suppress the proinflammatory cytokines after hepatectomy.

In contrast to previous reports[20], there was no difference in mortality between two groups during hospital stay and the 6-month follow up. However, the short period of follow up in this study may be the culprit. In the study, we find that the length of hospital stay was shorter in the omega-3 fatty acid group than that of the control group, which is due to the reduction of infection, especially the reduction of pneumonia and increased rate of recovery of liver function.

Omega-3 fatty acid supplementation ameliorates liver function after hepatectomy in the present study. The recovery of liver function is significantly faster in the omega-3 fatty acid group than the control group, which is consistent with previous study in animal model[21]. ALT, AST, and TBIL recovered rapidly in the omega-3 fatty acid group, but no difference was observed in plasma albumin levels. However, all the patients received albumin infusion routinely after hepatectomy to keep plasma albumin higher than 30 g/L as our protocol. The improvement of clinical outcome could be explained by the influence of omega-3 fatty acid on immune function especially through suppressing the production of proinflammatory cytokines such as IL-6 and TNF-α.

In conclusion, omega-3 fatty acid–supplemented TPN reduces the occurrence of infection mainly through a reduced incidence of pneumonia and increases the recovery rate of liver function after hepatectomy. Clearly, this will benefit patients after hepatectomy. These findings will provide a strong rationale for the use of omega-3 fatty acid-supplemented regimens in clinical practice.
